# The arene–alkene photocycloaddition

**DOI:** 10.3762/bjoc.7.61

**Published:** 2011-04-28

**Authors:** Ursula Streit, Christian G Bochet

**Affiliations:** 1 Department of Chemistry, University of Fribourg, Chemin du Musée 9, CH-1700 Fribourg, Switzerland

**Keywords:** benzene derivatives, cycloadditions, Diels–Alder, photochemistry

## Abstract

In the presence of an alkene, three different modes of photocycloaddition with benzene derivatives can occur; the [2 + 2] or *ortho*, the [3 + 2] or *meta*, and the [4 + 2] or *para* photocycloaddition. This short review aims to demonstrate the synthetic power of these photocycloadditions.

## Introduction

**Scheme 1:**
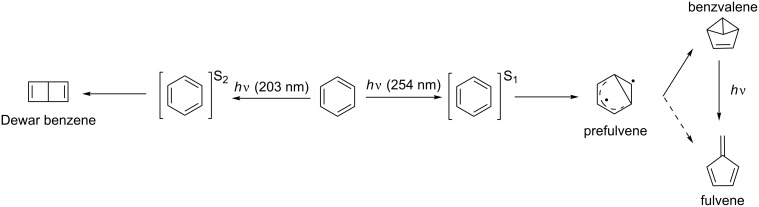
Photochemistry of benzene.

Photocycloadditions occur in a variety of modes [1]. The best known representatives are undoubtedly the [2 + 2] photocycloaddition, forming either cyclobutanes or four-membered heterocycles (as in the Paternò–Büchi reaction), whilst excited-state [4 + 4] cycloadditions can also occur to afford cyclooctadiene compounds. On the other hand, the well-known thermal [4 + 2] cycloaddition (Diels–Alder reaction) is only very rarely observed photochemically (vide infra). Photocycloadditions are not limited to simple alkenes, and even the thermally very stable benzene core has been shown to become quite reactive upon excitation with photons. It can be converted into benzvalene [2–3] and fulvene when excited to its first excited-state, or to Dewar benzene [4–5] via excitation to its second excited-state (Scheme 1) [6].

**Scheme 2:**
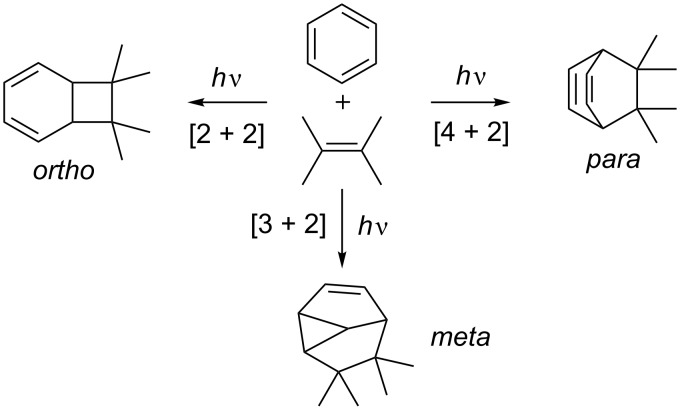
Three distinct modes of photocycloaddition of arenes to alkenes.

In the presence of an alkene, three different modes of photocycloaddition with benzene derivatives can occur, viz. the [2 + 2] or *ortho*, the [3 + 2] or *meta*, and the [4 + 2] or *para* photocycloaddition (Scheme 2). The descriptors *ortho*, *meta* and *para* only indicate the connectivity to the aromatic ring, and do not have any implication with regard to the reaction mechanism. This article aims at giving a didactic insight in the synthetic power of these photocycloadditions. While the field of *meta* photocycloadditions has been the focus of a series of excellent reviews, the *ortho* and *para* variants have only been reviewed in a more broad fashion.

In this article we will first discuss the *meta*, *ortho* and *para* modes, which we will call classical photocycloadditions of alkenes with arenes. The last part includes a related class of cycloadditions with arenes, where the aromaticity on the final compounds is restored. In this review, we will call this class “non-classical” photocycloadditions.

## Review

### Classical photocycloaddition of alkenes to arenes

While the *meta* photocycloaddition of benzene is very well documented in the literature and has been used many times in organic synthesis, the *ortho* and particularly the *para* photocycloadditions have not received as much attention since both types occur rarely and are usually low yield reactions. However, also in these two cases, the complexity of the products is considerably increased with respect to that of the reactants, as a new ring and up to four new stereocenters are formed.

The first “classical” [2π + 2π] photocycloaddition of benzene was described by Angus and Bryce-Smith in 1959 [7]. However, Ayer, Bradford and Büchi had obtained similar *ortho* products some four years earlier and recorded their findings in a patent [8]. The *meta* photocycloaddition was discovered in 1966 independently and almost simultaneously by Wilzbach and Kaplan [9] at Argonne, and by Bryce-Smith, Gilbert and Orger [10] at Reading. The *para* mode was the last to be discovered fifteen years later, again by Wilzbach and Kaplan [11]. Subsequently, considerable effort has been invested in an attempt to understand and further develop benzene photocycloadditions. This has resulted in some very spectacular results, among which is the application of the *meta* photocycloaddition in the total synthesis of natural products, particularly by Wender [12]. The field has been reviewed on several occasions mainly by Cornelisse [13], Mattay [14–15], Wender [16], Hoffmann [17] and de Keukeleire [18] with a focus on the *meta* mode.

#### Excited-states involved

Photocycloadditions of arenes with alkenes are usually triggered by the photoexcitation of the arene moiety. If the first excited singlet state of benzene is involved (^1^B_2u_, excited at 254 nm in a electric dipole forbidden transition, ε = 20.4), only the *meta* mode is allowed to occur in a concerted fashion according to molecular orbital symmetry rules [19–20]. Concerted *ortho* and *para* photocycloadditions of olefins are forbidden to occur from the first excited-state, but they are formally allowed from the second excited-state of benzene (^1^B_1u_). The fact that *ortho* and *para* cycloadducts are nevertheless observed to be formed can be explained if charge transfer processes are invoked, if reaction occurs in a non-concerted fashion (where the Woodward–Hoffmann rules do not apply), or from the second singlet excited-state. However, more recent computational work using a VB description of the structures has shown that such cycloadditions can take place from the S_1_ excited-state without barrier through a conical intersection, which is common to all three cycloadducts [21], at least for the benzene/ethylene system.

Quenching and sensitizing experiments were carried out to elucidate the spin state of the excited species involved. Mattay has shown that the quantum yield of the photocycloaddition of 1,3-dioxoles with benzene is halved for all three occurring modes (*ortho*, *meta* and *para*) if the reaction is carried out under an atmosphere of xenon rather than argon [22]. Xenon accelerates the singlet–triplet intersystem crossing by the heavy-atom effect, and thus decreases the singlet excited-state lifetime of the arene. Mattay considered this as direct proof that the addition of benzene to 1,3-dioxoles takes place primarily via the singlet excited-state of benzene. Ferree et al. had earlier provided evidence that the *meta* photocycloaddition occurs from the singlet excited-state; they observed that 6-phenyl-2-hexene undergoes cis–trans isomerization upon sensitizing with acetone and benzophenone, whereas the photocycloaddition to the *meta* product could only be triggered via direct irradiation [23].

#### Mode selectivity

Photocycloadditions are usually atom-economical, meaning that all atoms of the starting material end up in the final compound. Furthermore, photocycloadditions of alkenes with arenes are “step-economical”, as the complexity can be increased considerably in a single step. With these unprecedented possibilities to efficiently create diverse molecules with high complexity, synthetic applications should abound. One should, however, keep in mind, as Wender wrote some years ago, that*: “Any reaction with the potential to produce a highly complex product also has the potential to provide a highly complex product mixture, if its selectivity is not controlled”* [12]. Control of mode-, regio- and stereoselectivity is absolutely crucial if one aims at obtaining defined photocycloadducts in relevant yields.

The mode selectivity is predicted by the electronic properties of the two reaction partners involved. The (simplified) Weller equation determines whether or not electron transfer will occur from an excited-state (Equation 1) [24–25]. For the reaction of an excited aromatic moiety with an alkene, this means that if the change in free energy, determined by the Weller equation, is negative.

[1]
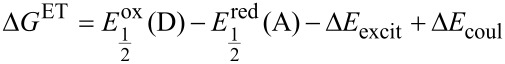


with Δ*G*^ET^ = free enthalpy of the radical ion pair formation, Δ*E*_excit_ = excitation energy of the chromophore, Δ*E*_coul_ = coulomb interaction energy of the radical ions and 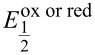 = half wave potential of donor and acceptor.

**Scheme 3:**
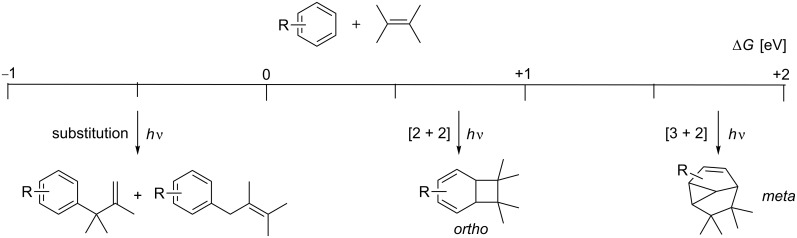
Mode selectivity with respect of the free enthalpy of the radical ion pair formation.

If electron transfer pathways dominate, substitution reactions are found to prevail. However, if the electron transfer is endergonic, *ortho* photocycloaddition can be observed. Mattay also observed that when Δ*G*^ET^ rises above 1.5 eV, *meta* photocycloadditions prevail (Scheme 3) [24].

**Scheme 4:**
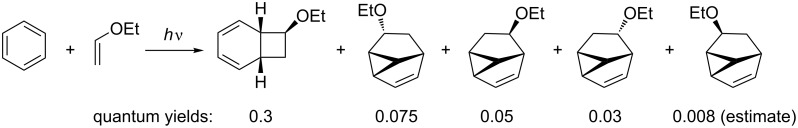
Photocycloaddition shows lack of mode selectivity.

However, these limits are not very sharp and a lack of mode selectivity may be observed in borderline regions (Scheme 4) [26]. These findings are consistent with the proposal that *ortho* photocycloadditions are the major reaction pathway when a certain degree of charge transfer is involved.

### *Meta* photocycloadditions

#### Mechanism

**Scheme 5:**
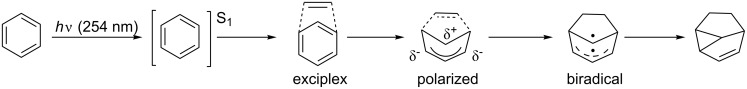
Mechanism of the *meta* photocycloaddition.

The *meta* photocycloaddition reaction was extensively reviewed in 1993 by Cornelisse [13], who gave also a summary of mechanistic suggestions and debates up to that date. The now commonly accepted mechanism of this reaction involves the excitation of the benzene moiety to its first excited-state (^1^B_2u_) and subsequent formation of an exciplex with the alkene moiety (Scheme 5).

**Scheme 6:**
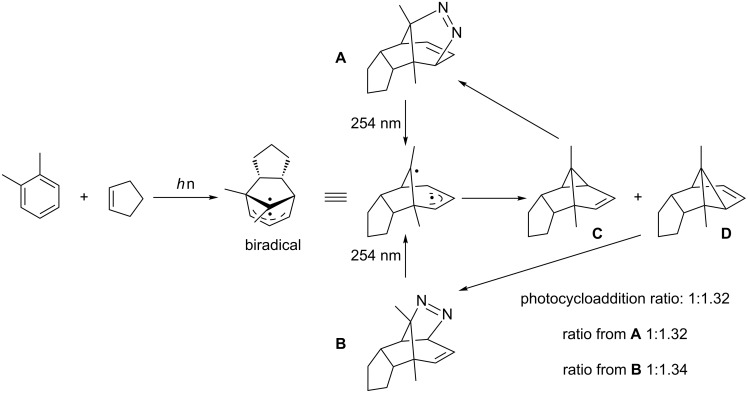
Evidence of biradiacal involved in *meta* photocycloaddition by Reedich and Sheridan.

The occurrence of such exciplexes has been detected by emission spectroscopy [22,27]. The two new sigma C–C bonds are formed concertedly from the exciplex. The reaction is thought to proceed through a slightly polarized intermediate, which explains the observed regioselectivity when the arene is disymmetrically substituted. While the bridging carbon atom is slightly positively charged, the termini of the allylic moiety carry a partial negative charge. It was shown that the formation of the sigma bonds is the rate determining step, as isotope effects were only observed when the addition occurred directly at the deuterium-substituted site [28]. On the other hand, it was also proposed that a biradical intermediate is involved in this photocycloaddition mechanism. Nevertheless, all attempts to trap a biradical or a zwitterionic intermediate have so far been unsuccessful. A very clever plan to test whether a biradical intermediate is indeed involved was carried out by Reedich and Sheridan [29]: By incorporating a diazo group into the last formed bond of the cyclopropyl ring in the *meta* photocycloadduct [30], they would be able to see whether the biradical formed by the extrusion of nitrogen gave the same products as the *meta* photocycloaddition (Scheme 6).

This was indeed the case, as very similar ratios of the two distinct stereoisomers **C** and **D** were found to be formed from the diazo compounds **A** and **B** as well as from the photocycloaddition of *o*-xylene to cyclopentadiene. This finding seems to indicate that a biradical structure is indeed involved in the *meta* photocycloaddition pathway; however, whether this biradical is an intermediate with a well-defined half-life or whether it is only just a transitory species remains so far unclear [31].

#### Regioselectivity

The *meta* photocycloaddition reaction creates a compound containing up to six new stereocenters in a single step from planar achiral starting materials. If substituents are added to the arene and the complexity of the olefin is increased, a great diversity of products can be created. However, not all of these products are accessible via such a photocycloaddition, as many possibilities can be ruled out due to the intrinsic selectivity of the reaction.

**Scheme 7:**
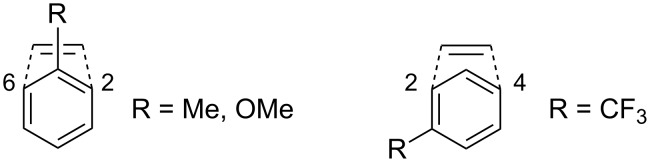
Regioselectivity with electron withdrawing and electron donating substituents.

There are two distinct regioselectivities involved in the *meta* photocycloaddition. The first is with regard to the substitution pattern on the aromatic ring. The influence of electron donating and electron withdrawing substituents on the reactivity is mainly on the polarized intermediate shown in Scheme 5. While electron donating substituents are usually known to direct the addition towards the 2,6-mode, they therefore end up on the positively polarized one-carbon bridge, whilst electron withdrawing substituents trigger a 2,4-addition and end up at one of the negatively polarized carbon atoms on the three-membered bridge (Scheme 7) [13,18].

**Scheme 8:**
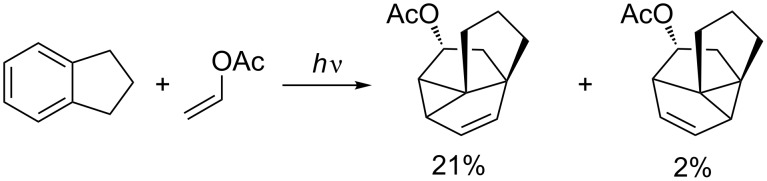
Closure of cyclopropyl ring affords regioisomers.

A second possibility to obtain different regioisomers occurs in the last step of the reaction. The recombination of the biradical may afford regioisomers when unsymmetrical olefins or additional substituents on the aromatic moiety are involved (Scheme 8).

However, *meta* photocycloadditions usually show a lack of regioselectivity at this stage of reaction, and thus mixtures of compounds are often isolated [32].

#### Stereoselectivity

**Scheme 9:**
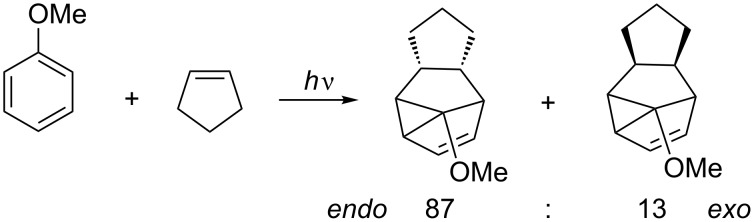
*Endo* versus *exo* product in the photocycloaddition of pentene to anisole [33].

If the olefin contains substituents, an issue of stereoselectivity is added to the regioselectivity. The addition can either occur from the *exo* or *endo* exciplex to the aromatic moiety (Scheme 9).

The observed preference for *endo* photocycloaddition [34–36] has been rationalized by secondary orbital overlap, which supports attack on the olefin from the *endo* position [37].

**Scheme 10:**
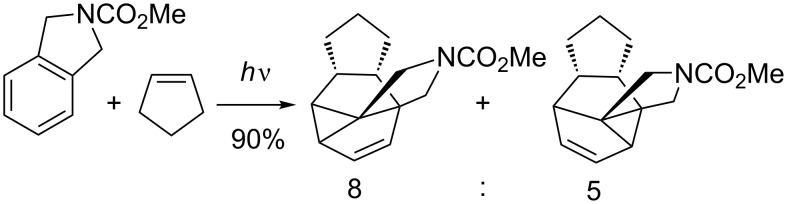
Regio- and stereoselectivity in the photocycloaddition of cyclopentene with a protected isoindoline.

An excellent example for such intermolecular selectivity was published a few years ago by Piet et al. (Scheme 10) [38].

They observed a very selective *endo* 1,3-photocycloaddition across the substituent on the aromatic moiety in the reaction of cyclopentene with a protected isoindoline. This reaction has been reported to produce very high yields; however, the lack of regioselectivity in the biradical cyclopropane formation gives the two regioisomers in a ratio of 8:5.

#### Intramolecular reactions

The first intramolecular *meta* photocycloaddition was reported in 1969 by Morrison and Ferree [39]. The intramolecular reaction was shown to be very efficient when three atoms tethers were employed, but the quantum yield drops considerably when four atoms tethers are involved [40], apart from some exceptional cases when the freedom of the tether is restricted and a suitable conformation can be found [41]. The influence of the incorporation of oxygen in the tether has been reviewed by de Keukeleire [42].

The selectivities mentioned above may change in intramolecular reactions. In this mode, the *endo* approach of the alkene cannot be achieved without introducing significant strain, which usually outweighs secondary orbital interactions, and thus *exo* rather than *endo* adducts are obtained [40].

**Scheme 11:**
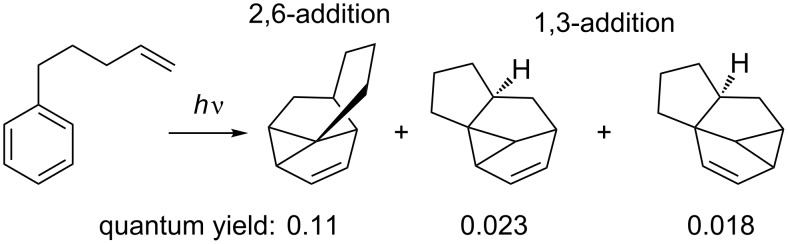
2,6- and 1,3-addition in intramolecular approach.

In the intramolecular mode, either 2,6- or 1,3-addition to the tether can be observed (Scheme 11) [43].

**Scheme 12:**
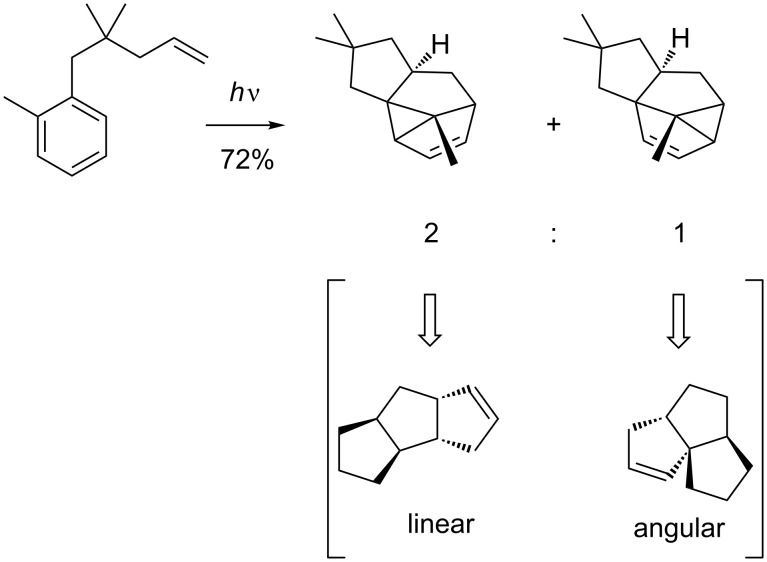
Linear and angularly fused isomers can be obtained upon intramolecular 1,3-addition.

1,3-Addition is usually observed selectively when electron donating groups are attached to the aromatic moiety *ortho* to the tether or if the olefin contains additional cis substituents; this is due to repulsion of the substituents on the olefin with the hydrogen atoms of the tether in the *exo*-addition which prevents the 2,6-addition mode. Intramolecular *meta* photocycloadditions that occur at positions 1,3 relative to the tether may give either a linear or an angular isomer (Scheme 12) [44].

Calculations of the relative stabilities are a useful tool to predict the selectivity. If no additional substituents are present, the linear compounds are slightly more stable [40]. However, some examples show that this selectivity can be changed [45]. The linear and angular isomer names are derived from the opening of the cyclopropane ring to afford linearly and angularly fused triquinanes.

#### Asymmetric *meta* photocycloadditions

**Scheme 13:**
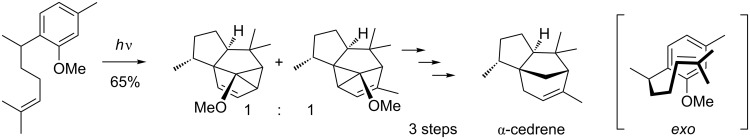
Synthesis of α-cedrene via diastereoselective *meta* photocycloaddition.

The *meta* photocycloaddition has the potential to convert planar (thus achiral) molecules into three-dimensional chiral molecules. It is therefore not surprising that many attempts have been made to render this process enantioselective. The main strategy in this perspective is the introduction of a chiral center directly on the tether in an intramolecular photocycloaddition, and to carry out the reaction in a diastereoselective way. The best known example of this approach was implemented in the total synthesis of (±)-α-cedrene published by Wender et al. in 1981 (Scheme 13) [46].

**Scheme 14:**
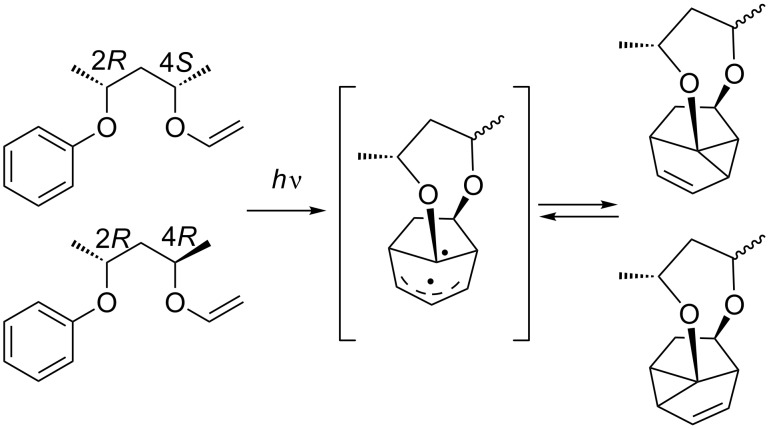
Asymmetric *meta* photocycloaddition introduced by chirality of tether at position 2.

Following this very elegant and highly efficient synthesis, the *meta* photocycloaddition attracted much interest in the field of total synthesis of natural products. In addition, studies aimed at understanding and predicting the diastereoselectivity of this photocycloaddition were undertaken. For example, the group of Sugimura has shown that chiral 2,4-pentanediol tethers are very efficient for face recognition (Scheme 14) [47–48].

The chirality at the position 2 of the tether completely directs the diastereoselectivity. Compounds having a 2*R* configuration react uniquely at the *si* face of the vinyl group, leading to the *R* configuration at the newly formed stereocenter. However, the reaction lacks regioselectivity in the formation of the cyclopropane ring, and both regioisomers are obtained. A similar approach has been published by Morales et al. who observed diastereoselectivity by the introduction of a chiral center α to the aromatic moiety on the tether [49]. Some diastereoselective approaches where the chiral induction is at position 3 of the tether have also been reported [50–51]. The diastereoselectivity observed is then due to steric interactions [51].

**Scheme 15:**
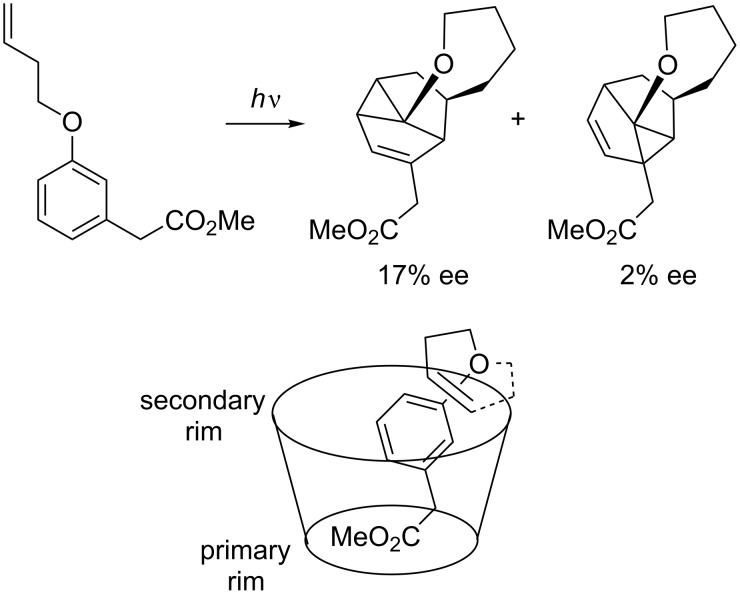
Enantioselective *meta* photocycloaddition in β-cyclodextrin cavity.

A completely different approach to an asymmetric *meta* photocycloaddition was made by Van der Eycken. He showed that *meta* photocycloaddition carried out in an chiral cavity (β-cyclodextrin) affords the photocycloaddition product in 17% ee (Scheme 15) [52].

#### Further diversification

**Scheme 16:**
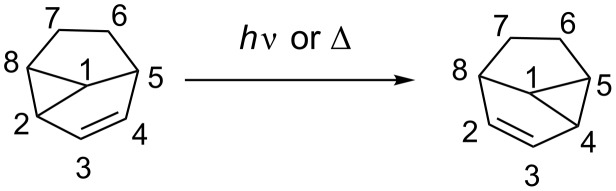
Vinylcyclopropane–cyclopentene rearrangement.

*Meta* photocycloaddition can be considered as very regio- and stereoselective if the appropriate substituents are chosen. The only reaction step notoriously lacking regioselectivity is the closing of the cyclopropane ring. Therefore, in many examples, the isolation of the two regioisomers of the *meta* photocycloaddition is described. However, a possibility to interconvert one compound into the other by thermal [53] as well as photochemical [47,54] means was found (Scheme 16).

**Scheme 17:**
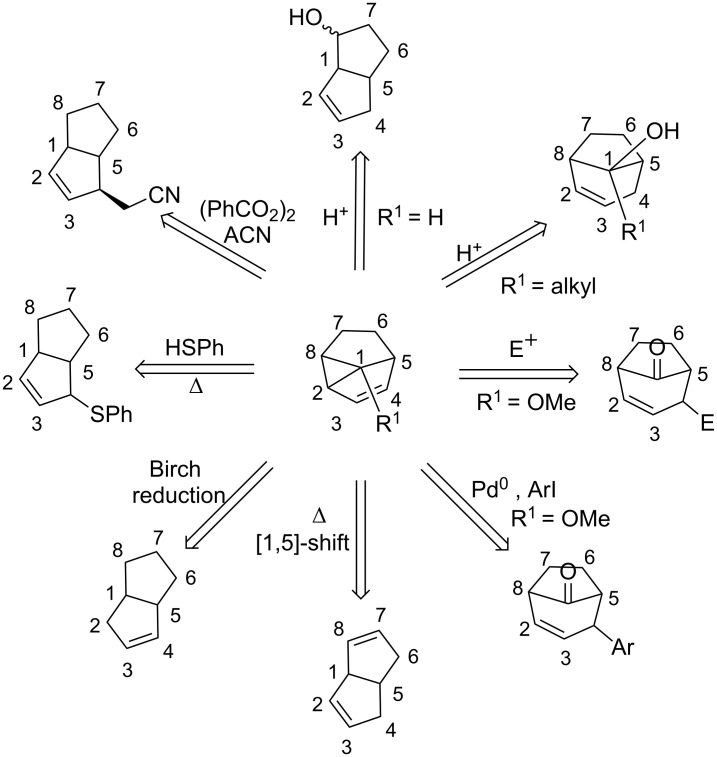
Further diversification possibilities of the *meta* photocycloaddition product.

The numerous applications of *meta* photocycloaddition in total syntheses show that this reaction can be a very efficient tool to access polycyclic natural compounds. However, the cores directly obtained are usually not useful as such, and cleavage of the cyclopropyl ring must be carried out to access naturally occurring polycyclic systems. Many investigations have already been achieved by different research groups to diversify the molecules accessible by this synthetic route: The cyclopropyl ring of the *meta* product is opened to give either bicyclo[3.3.0]octane or bicyclo[3.2.1]octane derivatives (Scheme 17).

Acidolysis of *meta* photocycloaddition compounds has been studied by Gilbert [55]. He observed that the cyclopropane ring opens upon protonation of the double bond. If the *meta* photocycloaddition product contains a substituent at position 1, ring opening affords bicyclo[3.2.1]octane derivatives, whilst if no substituent is present at position 1, a hexahydropentalene is the preferred product of ring opening. Other electrophiles also have the tendency to add to the double bond. Wender has shown in his total synthesis of (±)-α-cedrene, that bromine readily adds and triggers the opening of the cyclopropane ring [46]. Rearrangement of the resulting allylic bromide to the more stable regioisomer at this stage occurs readily and debromination can be achieved on treatment with tributyltin hydride. Penkett has shown that 3-chloroperbenzoic acid (*m*-CPBA) can take the role of an electrophile in the epoxidation of the double bond [56] and also demonstrated that the *meta* photocycloaddition products containing a methoxy group at position 1 can be converted in a Heck-type reaction [57–58]. The intermediate palladium σ-complex, formed by insertion, opens the cyclopropane ring and arylated bicyclo[3.2.1]octane derivatives are obtained. Thermolysis of the *meta* photocycloaddition product results in [1,5]-sigmatropic shifts. The shift can either be a shift of a hydrogen [54] or a carbon [59] atom to afford tetrahydropentalenes.

There are also radical approaches to the opening of the cyclopropyl ring. Under Birch reaction conditions, the cyclopropane ring can be reduced to the hexahydropentalene [45,60–61]. Radicals can also add to the double bond and induce the opening of the cyclopropane ring; for example, thiophenoxy radicals have been found to be very efficient for such reactions [44,62–63]. Another possibility to react radicals with *meta* photocycloadducts was demonstrated by Wender et al.*,* who showed that acetonitrile radicals can be added to the olefin and open the cyclopropane ring to yield a hexahydropentalene [64].

#### Application in total synthesis

There are several reviews that describe a large number of applications of the *meta* photocycloaddition to the total synthesis of complex polycyclic molecules. A recent publication by Chappell and Russell discusses comprehensively and in detail many examples from α-cedrene and the following 25 years [40]. We will therefore focus on the most recent examples of total synthesis applications.

Several times, Fenestranes have been the targets of total synthesis by *meta* photocycloaddition reactions [65]. Penkett, described very recently an access to fenestranes in a single step (not counting the one-step preparation of the substrate) [66].

**Scheme 18:**
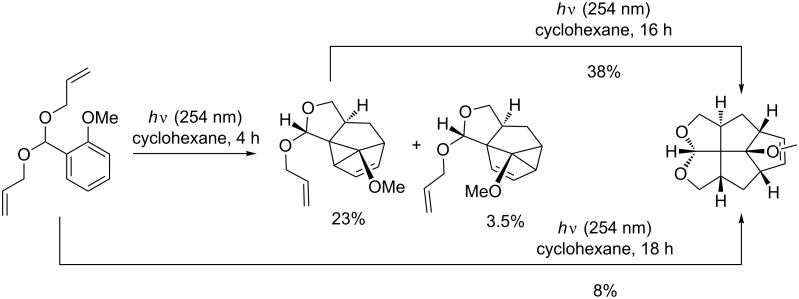
Double [3 + 2] photocycloaddition reaction affording fenestrane.

The reaction could also be carried out in a two step sequence. He observed that the starting material undergoes a selective 1,3-addition to one of the tethers. The subsequent cyclopropane ring closure gives mainly the linear fused *meta* product in 23% yield. The *meta* compounds and two rearranged *ortho* products were isolated from the reaction mixture in a combined yield of 60%. The major *meta* product can be converted into the fenestrane by further irradiation (Scheme 18). This second [3 + 2] addition reaction can be accelerated by the addition of sensitizers and could be quenched with piperylene. The proposed mechanism involves a homolytic cleavage of the cyclopropane ring to afford the linear triquinane biradical, which undergoes addition to the double bond. Wender achieved the total synthesis of fenestranes from *meta* photocycloaddition products some years earlier by cyclization using radicals in acetonitrile under reflux [67].

**Scheme 19:**
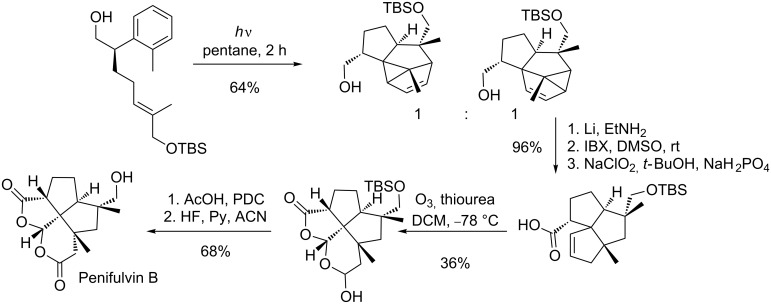
Total synthesis of Penifulvin B.

Another very recent application of the *meta* photocycloaddition has been published by Gaich and Mulzer [68]. They achieved the total synthesis of Penifulvin B and C in five steps after *meta* photocycloaddition (Scheme 19).

This first enantioselective total synthesis of the naturally occurring [5.5.5.6]fenestranes is highly efficient. The photocycloaddition occurs by an intramolecular 1,3-*exo* attack of the olefin to the aromatic moiety. The cyclopropane ring closure is, in this case, not regioselective at all, and leads to a 1:1 mixture of products. However, the undesired photocycloadduct can be converted to the desired compound via a thermal vinylcyclopropane–cyclopentene rearrangement. All obtained photocycloaddition compounds can be converted into the target by this means. The cyclopropane ring was opened by a Birch-type reduction. Oxidation of the primary alcohol and ozonolysis of the double bond afforded the hemiacetal after spontaneous cyclization. Subsequent oxidation and deprotection afforded the desired compound. In the same paper, the total synthesis of the naturally occurring epimer of Penifulvin B (Penifulvin C) was described, which was achieved by replacing the *trans-*olefin by the *cis-*olefin and following the same sequence of steps. One year earlier the same group accomplished the total synthesis of Penifulvin A via a *meta* photocycloaddition [69].

**Scheme 20:**
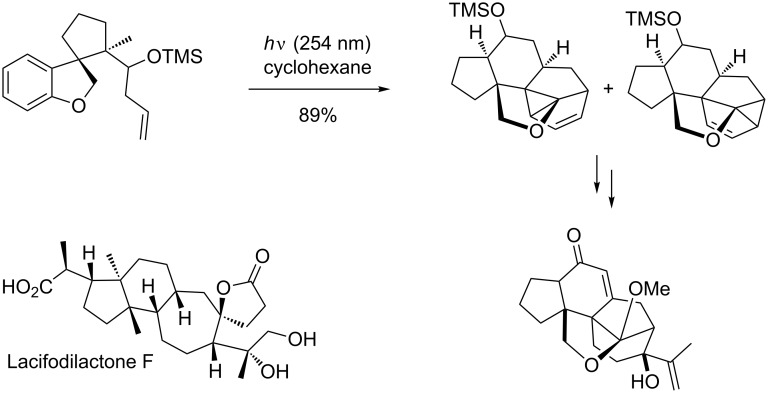
Towards the total synthesis of Lacifodilactone F.

Not only fenestranes are accessible from the *meta* photocycloaddition products. Wang and Chen have recently published an approach towards the core of Lancifodilactone F [70]. This triterpenoid contains three fused and one spiro cycle and is a very challenging target. Initial attempts to construct the fused seven-, six- and five-membered core were unsuccessful. Introduction of a trimethylsiloxy group on the tether and an oxygen substituent on the aromatic ring were crucial for the photocycloaddition to proceed in good yield (Scheme 20).

The additional tetrahydrofuran cycle helps to constrain the four unit tether. Only with these additional features does the cycloaddition step occur with a remarkable yield of 89%. The photocycloaddition occurs selectively 1,3-*exo* to the tether. The stereochemistry on the trimethylsiloxy substituent was shown to have no influence on the photochemistry, but it helped to direct the olefin group towards the arene moiety. The linear and angular fused compounds were elaborated to give a common intermediate containing the fused five-, six- and seven-membered ring pattern without further purification.

### *Ortho* photocycloaddition

**Scheme 21:**
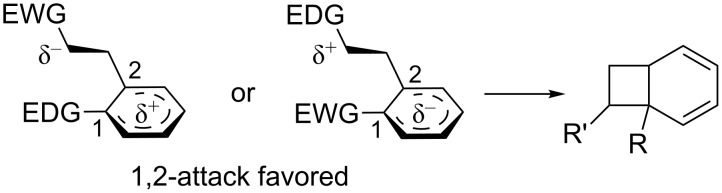
Regioselectivity of *ortho* photocycloaddition in polarized intermediates.

There is little data on the selectivity of the *ortho* photocycloaddition in the literature. However, Mattay discovered that the regioselectivity of the *ortho* photocycloaddition is dependent on the electronic properties of the two reaction partners [14]. He reported that 1,2-addition usually prevails when a high degree of charge transfer is involved in the exciplex (Δ*G*^ET^ below 0.5 eV). A substituent at position 1 stabilizes the charge which develops on the aromatic ring, and therefore favors the 1,2-ring closure of the intermediate (Scheme 21).

**Scheme 22:**
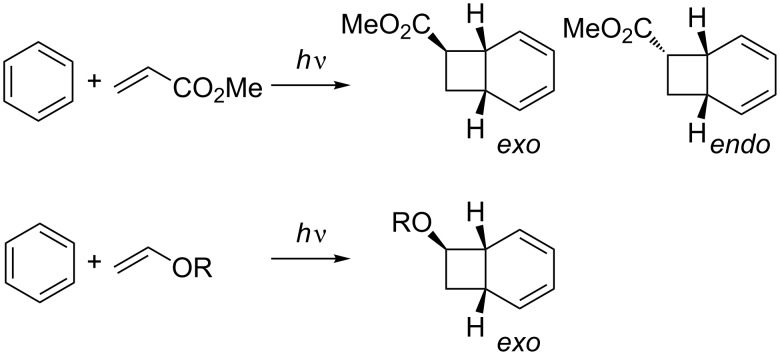
*Exo* and *endo* selectivity in *ortho* photocycloaddition.

This selectivity is not very high and as the degree of charge transfer decreases, the selectivity decreases. The role of CT is also supported by the fact that regioselectivity is usually higher in polar solvents where charge separation is favored [71–72]. Substituted olefins may add in an *exo* or *endo* fashion. While electron-deficient alkenes give mixtures of both possible products, electron-rich compounds show stereoselectivity towards the *exo* products (Scheme 22) [73].

**Scheme 23:**
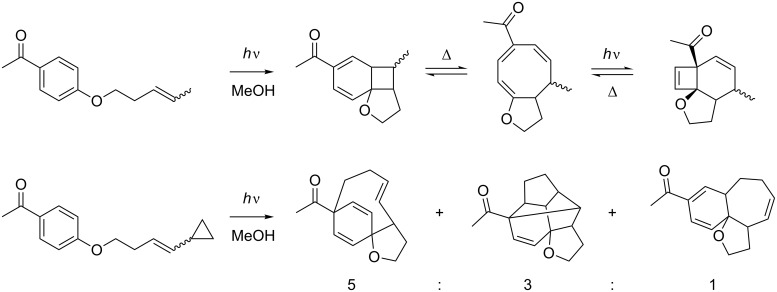
*Ortho* photocycloaddition of alkanophenones.

*Ortho* photocycloaddition can, however, also take place from the triplet excited-state by sensitization. The triplet excited-state is believed to have a higher biradical and less zwitterionic character [73]. *Ortho* photocycloaddition of triplet excited benzenes have been extensively studied by Wagner and his group. They investigated [2 + 2] photocycloadditions of alkanophenones, which occur from the triplet excited-state and via 1,4-biradicals [74–77]. The occurrence of such radicals has been proven by the insertion of a cyclopropyl radical trap in the olefin. Upon irradiation of this modified starting material, the *ortho* cycloadduct could no longer be isolated (Scheme 23) [78].

**Scheme 24:**
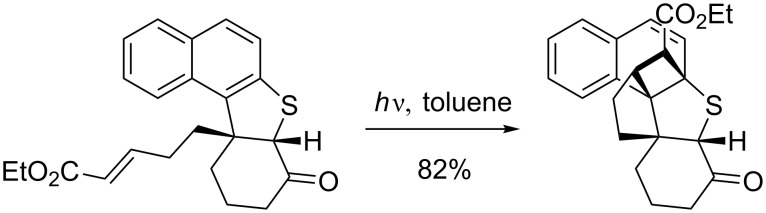
Photocycloadditions to naphtalenes usually in an [2 + 2] mode [79].

Naphthalenes are known to undergo preferably *ortho* photocycloadditions (Scheme 24), whilst higher arenes usually react with olefins in an [2 + 2], [4 + 2] or [4 + 4] cycloaddition mode.

**Scheme 25:**
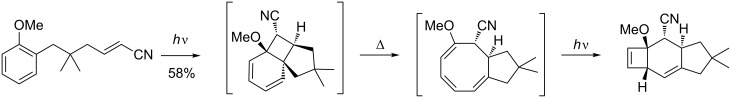
*Ortho* photocycloaddition followed by rearrangements.

The *ortho* photocycloaddition gives access to bicyclo[4.2.0]octanes which usually undergo thermal electrocyclic ring opening to yield eight-membered ring systems (Scheme 25) [73,80].

**Scheme 26:**
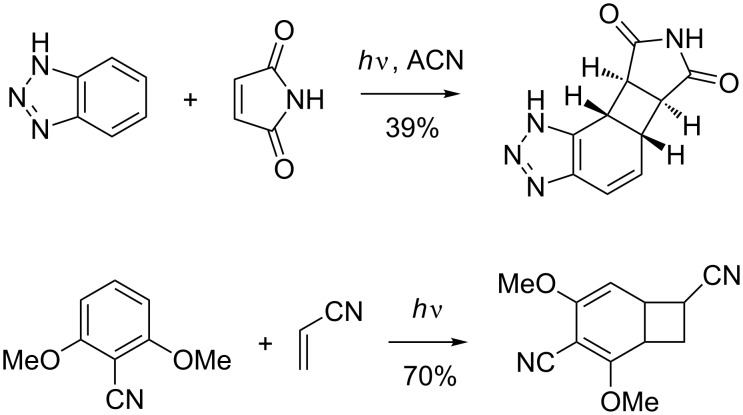
Stable [2 + 2] photocycloadducts.

Another possibility to further transform the intermediates are Diels–Alder cycloadditions of the dienes formed by the *ortho* photocycloaddition [81]. Furthermore, Scharf demonstrated that he could trigger a photochemical rearrangement to produce a *para* photocycloaddition product upon sensitization [82]. However, there also exists some examples where the [2 + 2] cycloadduct is stable enough to be isolated as such, and in good yield (Scheme 26) [83–84].

**Scheme 27:**
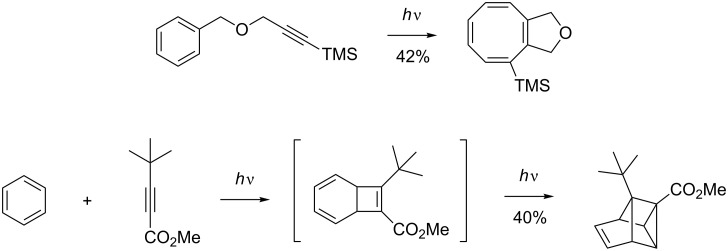
*Ortho* photocycloadditions with alkynes.

*Ortho* photocycloaddition is almost exclusively observed with alkynes. The photocycloaddition products usually undergo ring opening to cyclooctatetraenes [85] or to other rearrangement products [86] (Scheme 27).

**Scheme 28:**
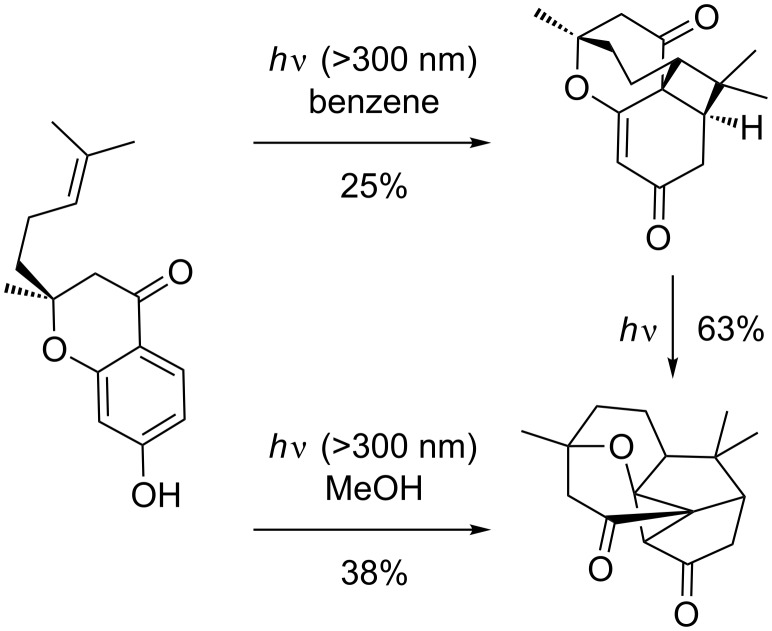
Intramolecular *ortho* photocycloaddition and rearrangement thereof.

*Ortho* photocycloadducts are known to be very unstable as such. However, if they have the possibility to stabilize by rearrangement, products can be isolated often in high yield. A very good example of this was published by Kalena et al. some years ago [87] who observed that 2-alkenyl-7-hydroxy-4-chromanone could undergo *ortho* photocycloaddition to afford the tetracyclic compounds shown in Scheme 28.

The initial enol tautomerizes to the α,β-unsaturated ketone, which can be isolated in moderate yield. Anisole photocycloaddition precursors did not afford similar tetracyclic compounds under the same irradiation conditions. However, under slightly acidic conditions (chlorinated solvents or additional *p*-toluenesulfonic acid) the photocycloaddition to the same compound could be restored.

Some years later, the same group reported that irradiation of the same starting material in methanol instead of benzene afforded the compound apparently formed by *meta* photocycloaddition [88]. However this *meta* photocycloaddition product has some issues. Addition occurs 2,6 across an electron-withdrawing substituent; this is unfavorable in a direct [1,3]-addition (due to the polarization in the polarized intermediate; see the chapter on "*Meta* photocycloadditions"). Furthermore, Kalena et al. could show that the compound is also formed upon irradiation of the *ortho* photocycloadditon product under slightly different irradiation conditions.

**Scheme 29:**
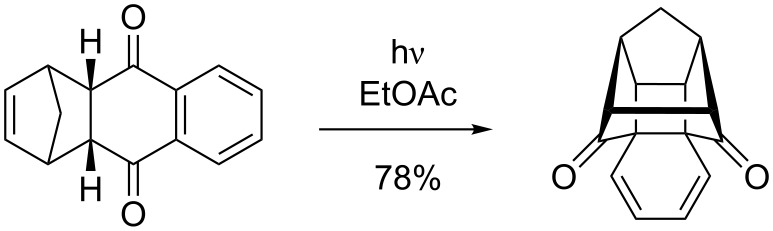
Intramolecular *ortho* photocycloaddition to access propellanes.

A very unconventional [2 + 2] photocycloaddition to afford caged polycyclic structures has been recently described (Scheme 29) [89–90].

By attaching the double bond very close to the aromatic moiety, the photocycloaddition leads to the desired cage compound in high yield. Surprisingly, the compound is fairly stable and can be recrystallized from hexane.

### *Para* photocycloaddition

**Scheme 30:**
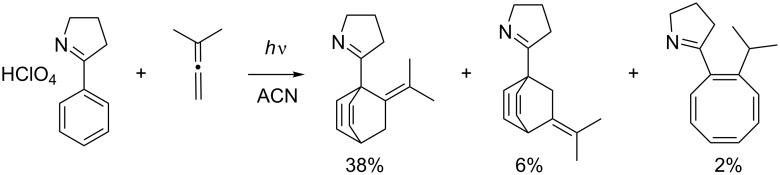
*Para* photocycloaddition with allene.

*Para* photocycloaddition giving access to bicyclo[2.2.2]octadiene systems by a formal Diels–Alder type reaction is very rare, and little is known about this mode. It is known that higher aromatics are more likely to undergo *para* cycloadditions [91], and that it is the main mode when allenes are involved [92]. A high yielding example of a *para* photocycloaddition with an allene has been published by Haddaway et al. (Scheme 30) [93].

If the irradiation is carried out in the presence of conjugated dienes or a second arene (usually higher aromatics), this photocycloaddition can also be carried out in a [4 + 4] mode to afford bicyclo[4.2.2]decatrienes and more complex homocycles [91,94–95].

**Scheme 31:**
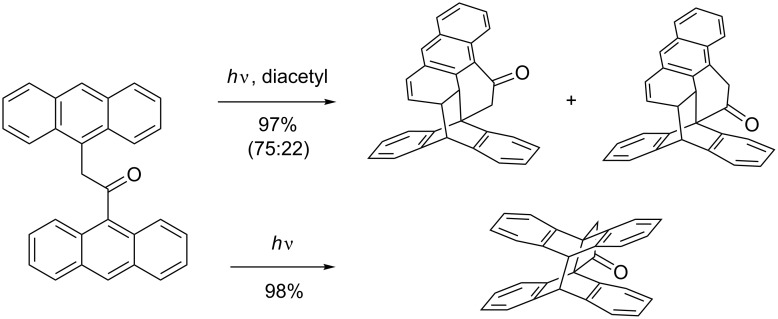
Photocycloadditions of dianthryls.

In the non-sensitized irradiation of dianthryls, the [4 + 4] photocycloaddition [91] pathway is usually observed; upon sensitizing in the triplet excited-state, the [4 + 2] mode prevails (Scheme 31). Such [4 + 4] photocycloadditions have also been observed intermolecularly with very high yields [96].

**Scheme 32:**
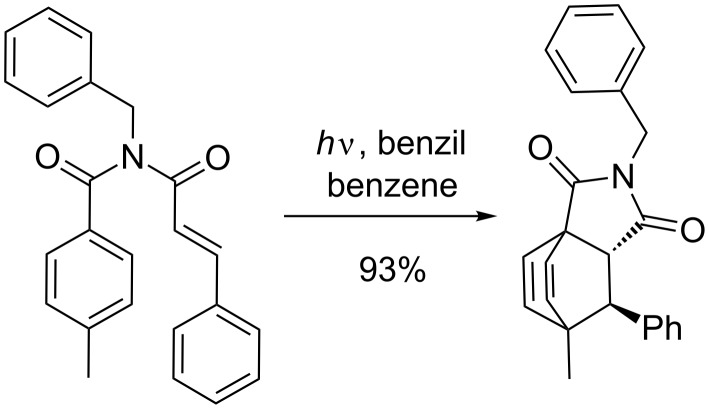
Photocycloaddition of enone with benzene.

There are few examples leading to *para* products in high yield. However, one example is the intramolecular photocycloaddition of a cinnamoylamide and a benzamide moiety (Scheme 32) [97]. This reaction is very efficient and leads to high yields of the bicyclo[2.2.2]octadiene derivative.

In this example, the cinnamoylamide is sensitized by benzil to its triplet excited-state. The proposed mechanism involves the reaction of the olefin with the ipso position of the aromatic ring affording a spiro biradical intermediate. Recombination of these radicals proceeds further until formation of the final compound. Some years later, Kohmoto showed that similar enamides linked to a naphthyl moiety underwent preferably *ortho* photocycloaddition if the naphthyl moiety is sensitized [98].

**Scheme 33:**
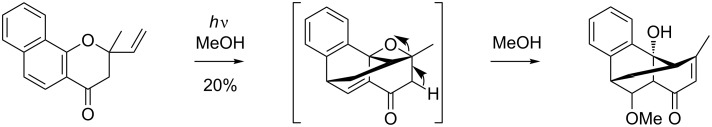
Intramolecular photocycloaddition affording multicyclic compounds via [4 + 2].

Irradiation of a naphthyl precursor containing only a two atom tether to the olefin afforded the *para* photocycloadduct (Scheme 33) [99].

However, Kalena et al. noted that the *para* product might also be derived from the *ortho* product upon further irradiation: The *para* product undergoes a sequence of ring opening/Michael addition of the solvent to give the final compound. However, neither the direct *ortho* nor the *para* product have been observed. The main changes from the previous papers of Kalena et al. were the replacement of the phenyl by a naphthyl group, and a two atom tether of the olefin. These two modifications are able to trigger different mode selectivity. As previously mentioned, the reaction with alkanophenones proceeds through a 1,4-biradical intermediate. The same reaction applied to this naphthyl derivative would lead initially to the formation of the four-membered ring. The five-membered ring will be preferred but the radical formed cannot recombine and fragments back to the starting material. Once the four-membered ring containing a primary exocyclic radical is formed, recombination either directly α to the carbonyl (construction of two fused cyclobutane ring systems) or delocalization of the radical to the *para* position can take place and the *para* product is formed.

### Non-classical photocycloadditions of alkenes with arenes

Not only can the benzene moiety of arenes undergo cycloadditions upon exposure to light but, depending on the substitution pattern, other reactive sites may be involved. Carbonyl groups are also known to undergo different types of photochemistry. Thus, benzophenones, acetophenones and benzaldehydes are not only used as sensitizers but can, under specific circumstances, also be directly involved in photochemical reactions to form new structures.

#### Formation of benzoxepines

**Scheme 34:**
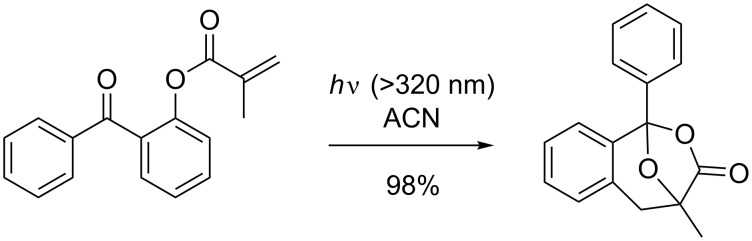
Photocycloaddition described by Sakamoto et al.

Sakamoto et al. reported that irradiation of *ortho* acylphenyl methacrylates can lead to photocycloaddition to afford benzoxepine structures in very high yield (Scheme 34) [100].

**Scheme 35:**
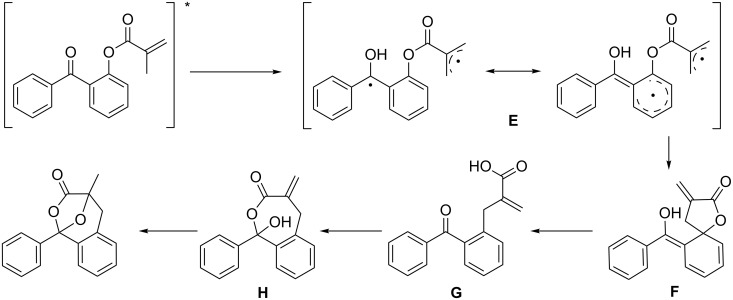
Proposed mechanism by Sakamoto et al.

This very unusual reaction involves the formation of a new aryl C–C bond and the loss of the aryl C–O bond, and is therefore clearly a rearrangement product. Furthermore, Sakamoto showed that this reaction was not limited to benzophenones, but also occurred with acetophenones, albeit in slightly lower yields. For this completely new reaction Sakamoto has proposed a mechanism involving a ζ-hydrogen abstraction to form a biradical intermediate (Scheme 35, **E**).

The resulting biradical cyclizes to form the spiro compound **F** upon recombination of the biradical. Re-aromatization affords the carboxylate **G**, which further attacks the carbonyl group. The alcohol intermediate **H** may cyclize by addition to the double bond to afford the final benzoxepine compound.

**Scheme 36:**
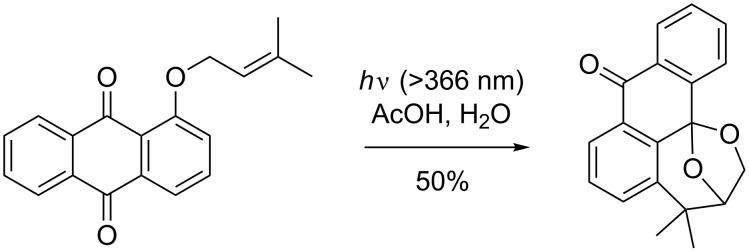
Photocycloaddition described by Jones et al.

In the same year, a slightly different reaction was reported by Jones et al. [101] who triggered the formation of unusual photocycloaddition products by irradiation of *ortho* allyloxy-substituted anthraquinones (Scheme 36).

**Scheme 37:**
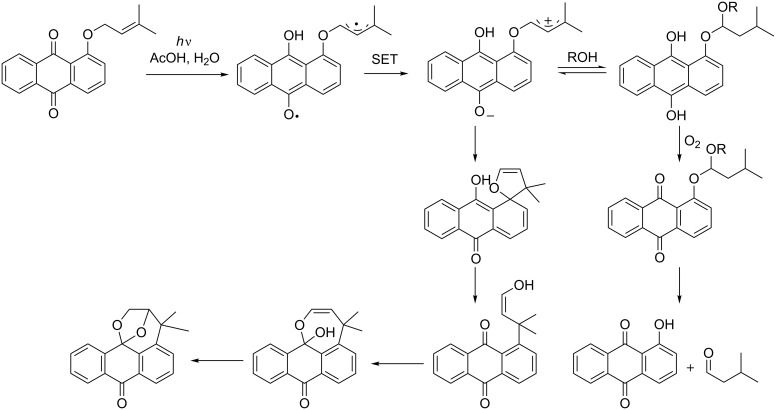
Proposed mechanism for the formation of benzoxepine by Jones et al.

During their study on the photo-release of bioactive aldehydes, Jones et al. discovered that, under anaerobic conditions, the dihydroquinone intermediate loses water to form the zwitterionic structure (Scheme 37).

From this intermediate, cyclization can take place to form a spiro compound; further re-aromatization to form the enol, lactolization and cyclization explains the formation of the benzoxepine structure [101].

**Scheme 38:**
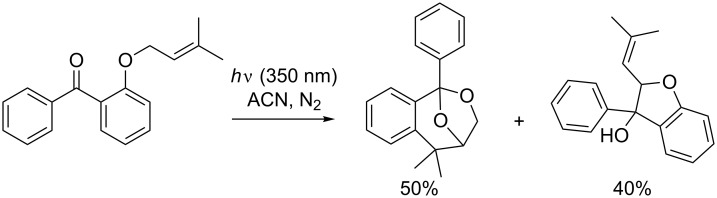
Photocycloaddition observed by Griesbeck et al.

Griesbeck et al. reported the formation of benzoxepines from the benzophenone analogue upon irradiation at slightly lower wavelengths [102]. The formation of the compound was observed in 50% yield, along with a diastereoisomeric mixture of dihydrobenzofurans in 40% yield (Scheme 38). Analogues of the dihydrobenzofuran formed upon irradiation of *ortho*-alkyloxyphenyl ketones have already been described in the literature and the reactions are known to take place via a δ-hydrogen abstraction by the ketone triplet, followed by cyclization of the 1,5-biradical intermediate [103].

**Scheme 39:**
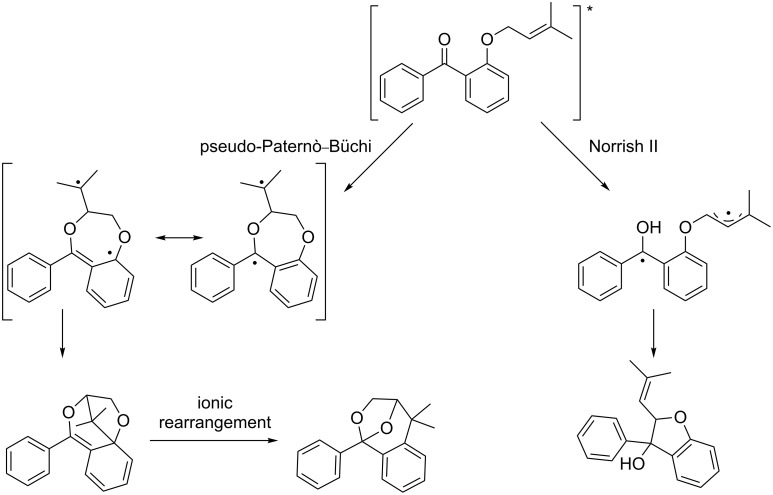
Mechanism proposed by Griesbeck et al.

Griesbeck et al. investigated the mechanism for this photocycloaddition, as he suggested that the mechanism proposed by Jones is unlikely, because the regiochemistry of proton catalyzed addition of alcohols to enols or enol ethers has the opposite regiochemistry to that observed in the product [102]. Furthermore, an electron transfer intermediate was ruled out, as the reaction is not thermodynamically feasible according to the Weller equation. Therefore, he proposed that the benzoxepine structure is achieved via a pseudo-Paternò–Büchi pathway (Scheme 39), while the dihydrobenzofurans arise from a Norrish-type II reaction and cyclization.

Griesbeck supports his proposed mechanism by flash laser photolysis, where a long lived (some seconds) intermediate with an UV absorption band at 380 nm was observed. This absorption band fits well with TD-DFT calculations. He proposes that re-aromatization of this intermediate takes place via a zwitterionic species or through a proton catalyzed pathway.

**Scheme 40:**
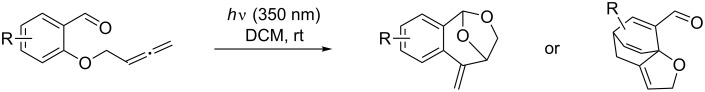
Intramolecular photocycloaddition of allenes to benzaldehydes.

We recently found in our laboratories that the intramolecular photocycloaddition of allenylated salicylaldehydes affords a benzoxepine derivative and an apparent *para* photocycloadduct (Scheme 40) [104]. The product distribution is dependent on the substitution pattern of the aromatic core.

Introduction of bulky *tert*-butyl substituents at positions 3 and 5 on the aromatic ring yields up to 94% of the *para* photocycloaddition product, while other substituents gave yields of up to 44% of the benzoxepine compound. Pericyclic reaction mechanisms for these two photocycloadditions have been proposed, but no hard evidence has so far been obtained. The mechanism of this unprecedented reaction is currently under investigation in our laboratory.

## Conclusion

In summary, we have described in this overview the applicability of the intriguing photocycloaddition of olefins with arenes. These reactions have been shown to afford compounds with a high increase in complexity in only one reaction step. We have discussed the diverse selectivities of the reaction, mechanisms as well as further modifications, and some of the most recent applications in total synthesis. Thus, while *meta* photocycloadditions have been exploited for over thirty years, *ortho* and, in particular, *para* photocycloadditions are uncommon and have consequently been less investigated. We have discussed these last two modes, which were exemplified by a few high yielding examples. Finally, we have reviewed the use and the reaction mechanism of the photocycloaddition of carbonyl substituted aromatics: Irradiation of *ortho* allyloxy, acrylic or allenyloxy substituted anthraquinones, benzophenones or benzaldehydes indeed give potentially interesting benzoxepines. There is little doubt that arene photochemistry will continue to help the synthetic chemist to assemble complex and challenging targets in the coming years.

## References

[1] Hoffmann N (2008). Chem Rev.

[2] Wilzbach K E, Ritscher J S, Kaplan L (1967). J Am Chem Soc.

[3] Kaplan L, Willzbach K E (1968). J Am Chem Soc.

[4] Van Tamelen E E, Pappas S P (1962). J Am Chem Soc.

[5] Van Tamelen E E, Pappas S P (1963). J Am Chem Soc.

[6] Harman P J, Kent J E, ODwyer M F, Griffith D W T (1981). J Phys Chem.

[7] Angus H J, Bryce-Smith D (1959). Proc Chem Soc, London.

[8] Ayer D E, Bradfort N H, Büchi G H (1957). 1-Cyanobicyclo[4.2.0]octa-2,4-dienes and their synthesis.

[9] Wilzbach K E, Kaplan L (1966). J Am Chem Soc.

[10] Bryce-Smith D, Gilbert A, Orger B H (1966). Chem Commun.

[11] Wilzbach K E, Kaplan L (1971). J Am Chem Soc.

[12] Wender P A, Ternansky R, deLong M, Sigh S, Olivero A, Rice K (1990). Pure Appl Chem.

[13] Cornelisse J (1993). Chem Rev.

[14] Mattay J (1987). J Photochem.

[15] Mattay J (2007). Angew Chem, Int Ed.

[16] Wender P A, Dore T M, Horspool W M, Song P-S (1995). Intra- and Intermolecular Cycloadditions of Benzene Derivatives. CRC Handbook of Organic Photochemistry and Photobiology.

[17] Hoffmann N (2004). Synthesis.

[18] De Keukeleire D, He S-L (1993). Chem Rev.

[19] Bryce-Smith D (1969). J Chem Soc D.

[20] van der Hart J A, Mulder J J C, Cornelisse J (1995). J Photochem Photobiol, A.

[21] Clifford S, Bearpark M J, Bernardi F, Olivucci M, Robb M A, Smith B R (1996). J Am Chem Soc.

[22] Mattay J, Leismann H, Scharf H-D (1979). Mol Photochem.

[23] Ferree W, Grutzner J B, Morrison H (1971). J Am Chem Soc.

[24] Mattay J (1985). Tetrahedron.

[25] Müller F, Mattay J (1993). Chem Rev.

[26] Gilbert A, Taylor G N, Wahid bin Samsudin M (1980). J Chem Soc, Perkin Trans 1.

[27] Leismann H, Mattay J, Scharf H-D (1984). J Am Chem Soc.

[28] De Vaal P, Lodder G, Cornelisse J (1990). J Phys Org Chem.

[29] Reedich D E, Sheridan R S (1985). J Am Chem Soc.

[30] Sheridan R S (1983). J Am Chem Soc.

[31] Muller P (1994). Pure Appl Chem.

[32] Wender P A, Dreyer G B (1982). J Am Chem Soc.

[33] De Vaal P, Osselton E M, Krijnen E S, Lodder G, Cornelisse R (1988). Recl Trav Chim Pays-Bas.

[34] Ors J A, Srinivasan R (1977). J Org Chem.

[35] Merritt V Y, Cornelisse J, Srinivasan R (1973). J Am Chem Soc.

[36] Mattay J, Rumbach T, Runsink J (1990). J Org Chem.

[37] Houk K N (1982). Pure Appl Chem.

[38] Piet D, De Bruijn S, Sum J, Lodder G (2005). J Heterocycl Chem.

[39] Morrison H, Ferree W I (1969). J Chem Soc D.

[40] Chappell D, Russell A T (2006). Org Biomol Chem.

[41] Boyd J W, Greaves N, Kettle J, Russell A T, Steed J W (2005). Angew Chem, Int Ed.

[42] De Keukeleire D (1994). Aldrichimica Acta.

[43] Gilbert A, Taylor G N (1979). J Chem Soc, Chem Commun.

[44] Baralotto C, Chanon M, Julliard M (1996). J Org Chem.

[45] Wender P A, Von Geldern T W, Levine B H (1988). J Am Chem Soc.

[46] Wender P A, Howbert J J (1981). J Am Chem Soc.

[47] Sugimura T, Yamasaki A, Okuyama T (2005). Tetrahedron: Asymmetry.

[48] Hagiya K, Yamasaki A, Okuyama T, Sugimura T (2004). Tetrahedron: Asymmetry.

[49] Calderon Morales R, Lopez-Mosquera A, Roper N, Jenkins P R, Fawcett J, García M D (2006). Photochem Photobiol Sci.

[50] Guo X-C, Chen Q-Y (1999). J Fluorine Chem.

[51] Timmermans J L, Wamelink M P, Lodder G, Cornelisse J (1999). Eur J Org Chem.

[52] Vízvárdi K, Desmet K, Luyten I, Sandra P, Hoornaert G, Van der Eycken E (2001). Org Lett.

[53] Doering W von E, Lambert J B (1963). Tetrahedron.

[54] Wender P A, Dreyer G B (1981). Tetrahedron.

[55] Fenton G A, Gilbert A (1989). Tetrahedron.

[56] Avent A G, Byrne P W, Penkett C S (1999). Org Lett.

[57] Penkett C S, Sims R O, French R, Dray L, Roome S J, Hitchcock P B (2004). Chem Commun.

[58] Penkett C S, Sims R O, Byrne P W, Kingston L, French R, Dray L, Berritt S, Lai J, Avent A G, Hitchcock P B (2006). Tetrahedron.

[59] Srinivasan R (1971). J Am Chem Soc.

[60] Wender P A, Ternansky R J (1985). Tetrahedron Lett.

[61] Coates R M, Ho J Z, Klobus M, Zhu L (1998). J Org Chem.

[62] Wender P A, Howbert J J (1983). Tetrahedron Lett.

[63] Wender P A, Dore T M (1998). Tetrahedron Lett.

[64] Wender P A, deLong M A (1990). Tetrahedron Lett.

[65] Keese R (2006). Chem Rev.

[66] Penkett C S, Woolford J A, Day I J, Coles M P (2010). J Am Chem Soc.

[67] Wender P A, Dore T M, deLong M A (1996). Tetrahedron Lett.

[68] Gaich T, Mulzer J (2010). Org Lett.

[69] Gaich T, Mulzer J (2009). J Am Chem Soc.

[70] Wang Q, Chen C (2008). Org Lett.

[71] Gilbert A, Yianni P (1981). Tetrahedron.

[72] Gilbert A, Yianni P (1982). Tetrahedron Lett.

[73] Coxon J M, Halton B (1987). Organic Photochemistry.

[74] Wagner P J (2001). Acc Chem Res.

[75] Wagner P J, Sakamoto M, Madkour A E (1992). J Am Chem Soc.

[76] Wagner P J, Nahm K (1987). J Am Chem Soc.

[77] Wagner P J, Nahm K (1987). J Am Chem Soc.

[78] Cheng K-L, Wagner P J (1994). J Am Chem Soc.

[79] Dittami J P, Nie X Y, Nie H, Ramanathan H, Buntel C, Rigatti S, Bordmer J, Decosta D L, Williard P (1992). J Org Chem.

[80] Nuss J M, Chinn J P, Murphy M M (1995). J Am Chem Soc.

[81] Mathew T, Tonne J, Sedelmeier G, Grund C, Keller M, Hunkler D, Knothe L, Prinzbach H (2007). Eur J Org Chem.

[82] Scharf H-D, Leismann H, Erb W, Gaidetzka H W, Aretz J (1975). Pure Appl Chem.

[83] Brooker-Milburn K I, Wood P M, Dainty R F, Urquhart M W, White A J, Lyon H J, Charmant J P H (2002). Org Lett.

[84] Al-Jalal N, Gilbert A (1990). Recl Trav Chim Pays-Bas.

[85] Pirrung M C (1987). J Org Chem.

[86] Hanzawa Y, Paquette L (1982). Synthesis.

[87] Kalena G P, Pradhan P, Banerji A (1992). Tetrahedron Lett.

[88] Kalena G P, Pradhan P, Banerji A (1999). Tetrahedron.

[89] Kotha S, Dipak M K (2006). Chem–Eur J.

[90] Kushner A S (1971). Tetrahedron Lett.

[91] Becker H-D, Hansen L, Andersson K (1986). J Org Chem.

[92] Bryce-Smith D, Foulger B E, Gilbert A (1972). J Chem Soc, Chem Commun.

[93] Haddaway K, Somekawa K, Fleming P, Tossell J A, Mariano P S (1987). J Org Chem.

[94] Yang N C, Libman J (1973). Tetrahedron Lett.

[95] Berridge J C, Bryce-Smith D, Gilbert A (1975). Tetrahedron Lett.

[96] Kurata H, Kyusho M, Nishimae Y, Matsumoto K, Kawase T, Oda M (2007). Chem Lett.

[97] Kishikawa K, Akimoto S, Kohmoto S, Yamamoto M, Yamada K (1997). J Chem Soc, Perkin Trans 1.

[98] Kohmoto S, Miyaji Y, Tsuruoka M, Kishikawa K, Yamamoto M, Yamada K (2001). J Chem Soc, Perkin Trans 1.

[99] Kalena G P, Pradhan P, Puranik V S, Banerji A (2003). Tetrahedron Lett.

[100] Nishio T, Sakurai N, Iba K, Hamano Y-I, Sakamoto M (2005). Helv Chim Acta.

[101] Brinson R G, Hubbard S C, Zuidema D R, Jones P B (2005). J Photochem Photobiol, A.

[102] Pérez-Ruiz R, Hinze O, Neudörfl J-M, Blunk D, Görner H, Griesbeck A G (2008). Photochem Photobiol Sci.

[103] Wagner P J, Meador M A, Park B-S (1990). J Am Chem Soc.

[104] Birbaum F, Neels A, Bochet C G (2008). Org Lett.

